# Clozapine Regulates Microglia and Is Effective in Chronic Experimental Autoimmune Encephalomyelitis

**DOI:** 10.3389/fimmu.2021.656941

**Published:** 2021-05-03

**Authors:** Ulaş Ceylan, Steffen Haupeltshofer, Laura Kämper, Justus Dann, Björn Ambrosius, Ralf Gold, Simon Faissner

**Affiliations:** Department of Neurology, Ruhr-University Bochum, St. Josef-Hospital, Bochum, Germany

**Keywords:** progressive multiple sclerosis, neuroprotection, microglia, iron, EAE (experimental autoimmune encephalomyelitis), clozapine

## Abstract

**Objective:**

Progressive multiple sclerosis is characterized by chronic inflammation with microglial activation, oxidative stress, accumulation of iron and continuous neurodegeneration with inadequate effectiveness of medications used so far. We now investigated effects of iron on microglia and used the previously identified neuroprotective antipsychotic clozapine *in vitro* and in chronic experimental autoimmune encephalomyelitis (EAE).

**Methods:**

Microglia were treated with iron and clozapine followed by analysis of cell death and response to oxidative stress, cytokine release and neuronal phagocytosis. Clozapine was investigated in chronic EAE regarding optimal dosing and therapeutic effectiveness in different treatment paradigms. Animals were scored clinically by blinded raters. Spinal cords were analyzed histologically for inflammation, demyelination, microglial activation and iron accumulation and for transcription changes of regulators of iron metabolism and inflammation. Effects on immune cells were analyzed using flow cytometry.

**Results:**

Iron impaired microglial function *in vitro* regarding phagocytosis and markers of inflammation; this was regulated by clozapine, reflected in reduced release of IL-6 and normalization of neuronal phagocytosis. In chronic EAE, clozapine dose-dependently attenuated clinical signs and still had an effect if applied in a therapeutic setting. Early mild sedative effects habituated over time. Histologically, demyelination was reduced by clozapine and positive effects on inflammation strongly correlated with reduced iron deposition. This was accompanied by reduced expression of DMT-1, an iron transport protein.

**Conclusions:**

Clozapine regulates microglial function and attenuates chronic EAE, even in a therapeutic treatment paradigm. This well-defined generic medication might therefore be considered as promising add-on therapeutic for further development in progressive MS.

## Introduction

Multiple Sclerosis (MS) is a multifactorial chronic-inflammatory disorder of the central nervous system, leading to neurodegeneration and chronic disability (1). While nowadays a broad spectrum of medications is available for the relapsing-remitting phase (RRMS) of the disease with differing efficacy and side effect profiles (2) it still remains challenging to tackle the progressive phase of the disease. Reasons for this are differing mechanisms of chronic inflammation with predominance of trapped inflammation behind the blood brain barrier (BBB) of cells of innate immunity such as microglia, release of iron, oxidative stress and cellular damage also including mitochondrial impairment – altogether fueling progressive neurodegeneration and clinically functional impairment (3, 4). Until now, only a limited number of medications have been FDA-approved for either (active) secondary progressive MS (SPMS; interferon-β1a or b, mitoxantrone, cladribine, siponimod) or primary progressive MS (PPMS; ocrelizumab) (5). Those medications are an important step to slow down progression but still have limited efficacy (interferons) or severe sideeffects (mitoxantrone); it therefore remains crucial to better understand and target pathomechanisms of progression to further improve therapy for those with progressive forms of MS.

To address this need, we and others have used systematic screening approaches to target features of progressive MS and identify protective medications. Approaches were directed to enhance remyelination (6) or reduce neurodegeneration by iron (7). Iron age-dependently accumulates in the CNS of progressive MS patients (8) and might amplify cellular damage by driving inflammation and generating reactive oxygen metabolites *via* the Fenton reaction (9). To address this mechanism, we conducted a high throughput screening and identified several orally available generic medications with presumably neuroprotective features (7). One of those medications was the atypic antipsychotic clozapine, which reduced iron-mediated neurotoxicity and prevented mitochondrial damage to neurons, reduced T cell proliferation, and showed antioxidative properties (7). We here set out to better understand effects of iron on microglia in culture and investigated clozapine both *in vitro* and in an animal model of progressive MS, chronic experimental autoimmune encephalomyelitis, in different therapeutic paradigms.

## Methods

### Cell Culture

#### HMC3 Cells

Microglia of the human microglial cell line 3 (HMC3) (10) were used as previously described (11). HMC3 cells were cultured in T75 flasks in Minimum Essential Medium (MEM, no glutamine) supplemented with 1% 10,000 units/ml penicillin/streptomycin, 1% glutamine (GlutaMAX Supplement; all Gibco, Life Technologies, Carlsbad, CA, USA) and 10% fetal bovine serum (FBS) (FBS Standard, Pan Biotech, Aidenbach, Germany). Cells with a confluence of 90% were split using Accutase (Invitrogen, Life Technologies, Carlsbad, CA, USA). For experiments, cells were cultured for at least 24 h in 96 well plates in a density of 20,000 cells/well (200,000 cells/ml). Cells were treated with iron sulfate (FeSO_4,_ Iron(II)) sulfate heptahydrate; Sigma-Aldrich, St. Louis, MO, USA) as previously described (7) and diluted in medium to achieve desired concentrations. Clozapine was prepared fresh and dissolved in DMSO 0.025%. After 24 h the cells were stained with Hoechst 33342 (6 µg/ml, Invitrogen) for 90 min and Propidium iodide (PI, 400 ng/ml; Invitrogen) for 15 min. Thereafter, cells were washed and four double-images were taken per well (Olympus BX51, Tokyo, Japan, 10x). The images were analyzed for cell particle number and area with macro instructions for ImageJ (National Institutes of Health, Bethesda, MD, USA). The values of PI^+^ images were divided by the corresponding values of Hoechst-images to determine relative cell death/apoptosis. The viability was measured with Calcein AM staining using a fluorescence plate reader at 530 nm (Infinite 200 Pro, Tecan Group AG, Männedorf, Switzerland).

#### N2a Cells

Neuro2a mouse neuroblastoma cells (N2a, Department of Neuroanatomy and Molecular Brain Research, Ruhr-University Bochum, Bochum, Germany) were cultured in T75 flasks in DMEM (DMEM, high glucose, GlutaMAX Supplement, Gibco) with 1% 10,000 units/ml penicillin/streptomycin and 5% FBS. Cells were split using trypsin-EDTA 0.5% (Gibco) at a confluency of 90%. For experiments, cells were detached, heated for 10 min in 90°C PBS and cooled down on ice for another 10 min to secure cell death.

#### Secretome Analysis

20,000 HMC3 cells/well were plated in a 96 well plate and incubated for 24 h, following medium change, treatment with clozapine for 1 h and stimulation with FeSO_4_ for another 24 h. Supernatants were harvested and stored at -80°C. Cytokines were analyzed using the Cytokine Cytometric Bead Assay (BD Biosciences, Franklin Lakes, NJ, USA) on a FACS Canto II (BD Biosciences) as previously described (12). Data were analyzed using the software FACS Cap Array v.3.0.

#### Oxidative Stress

Tert-Butyl hydroperoxide (t-BHP) was used to induce oxidative stress. 20,000 HMC3 cells/well were plated in a 96 plate and incubated for 24 h. Clozapine was added 1 h prior to addition of t-BHP in different concentrations between 50 µM and 800 µM following analysis using the MTT after 2 and 4 h. The absorbance was measured at 570 nm using a plate reader.

#### Phagocytosis Assay

HMC3 cells were plated at a density of 20,000 cells/well in 96 well plates. Dead N2a cells following heat treatment as indicated above were stained with PI (400 ng/ml) for 15 min. 50,000 stained and dead N2a cells were added to each well and incubated for 1 h. After 2 wash steps with cold PBS fluorescence intensity was measured at 535 nm/617 nm with a plate reader (Synergy H1, BioTek Instruments, Winooski, VT, USA; Tecan).

### Experimental Autoimmune Encephalomyelitis

All animal experiments were approved by the animal care committee of North Rhine-Westphalia, Germany (LANUV, no. 84-02.04.2017.A132). For all experiments, seven-week-old female C57BL/6J mice were used (Janvier Lab, Le Genest-Saint-Isle, France). Mice were housed under environmentally controlled conditions with constant temperature and a 12:12 h dark-light cycle under pathogen free environmentally controlled conditions. Mice had free access to chow and water *ad libitum.* Prior experiment start, mice were adapted to the environment for at least one week. Experimental autoimmune encephalomyelitis (EAE) was induced upon injection of an emulsion containing 500 µg/ml Myelin Oligodendrocyte Glycoprotein_35-55_ (MOG_35-55_) in Complete Freund’s adjuvant containing 2,000 µg/ml *Mycobacterium tuberculosis* as previously described (13). 50 µl emulsion was injected subcutaneously in each hind flank. Mice were injected with 200 ng Pertussis toxin dissolved in PBS on days 0 and 2 to induce blood-brain-barrier leakage. Animals were scored daily before administration of clozapine to rule out sedative effects according to a previously defined scoring scheme with the following scores: 0: no signs of disability; 1: tail paresis; 2: complete tail paralysis; 3: missing compensatory movements while walking; 4: ataxia; 5: moderate hind leg paresis; 6: complete paresis of one hind leg or stronger paresis of both hind legs; 7: paraplegia; 8: tetraparesis; 9: moribund; 10: death (13). Mice with a score of 7 were euthanized according to animal care guidelines. Before treatment initiation animals were randomized according to weight or according to the score in the therapeutic experiments. Animals were treated with clozapine prophylactically from day 0 or therapeutically by oral gavage as indicated in respective figure legends. Clozapine was solved in PBS.

#### Open-Field

The activity of animals was evaluated on a weekly basis using the open-field test. The test was performed in a quiet environment without disturbing stimuli. Activity was tracked for 15 min and analyzed regarding track, speed, activity time and rearing. To minimize the effect of habituation, two baseline measurements were performed before induction of EAE. The chamber was cleaned with 70% ethanol and water between each measurement to minimize disturbance by animal odor.

#### Explant

The EAE was terminated 12 h after the last administration of clozapine and mice were anesthetized with 120 mg/kg ketamine and 16 mg/kg xylazine. Blood samples were taken by intracardiac puncture and animals were subjected to PBS-perfusion. Spleens and lymph nodes (axillary, cervical and inguinal) were obtained for flow cytometry. Before fixation a small sample of the lumbar spinal cord was snap frozen for further PCR analysis. The remainder of spinal cords and brains were fixed in 4% buffered formalin. After fixation, the spinal cords were divided into cervical, thoracic and lumbar parts, put in cassettes, filled up with Frozen Section Medium NEG-50 (Sigma Aldrich, St. Louis, MO, USA) and placed immediately on dry ice following storage at a temperature of -20°C. Blood cells, lymph node cells and splenocytes were used for flow cytometry analysis.

### Flow Cytometry

Cells from lymph nodes and spleens were obtained by pressing them through 100 µm and 70 µm strainers and washing with cold PBS. Splenocytes and blood cells were put in Erythrocytes Lysing Buffer (150 mM NH_4_Cl, 10 mM KHCO_3_, 1 mM Triplex III) to eliminate erythrocytes. Cells were stained with primary antibodies (**Additional Table 1A**) and analyzed by flow cytometry using FACS Canto II (BD Biosciences, Franklin Lakes, NJ, USA). Data were analyzed using FlowJo (FlowJo X 10.0.7r2, Becton, Dickinson and Company, Ashland, OR, USA).

### Histology

Cryosections were stained with haematoxylin (Sigma Aldrich) and eosin 0.1% solution (Merck), anti-Iba-1 (FUJIFILM Wako Pure Chemical Corporation, Osaka, Japan; **Additional Table 1B**) and goat anti-rabbit-immunoglobulin-Alexa Fluor 568 (abcam, Cambridge, UK) to stain microglia, FluoroMyelin (Invitrogen) to stain myelin and acidified 20% potassium ferricyanide solution (Laborladen.de, Hüfingen, Germany) with DAB intensification (Merck) to stain ferrous iron. All images were merged (Image Composite Editor, Microsoft Corporation, Redmond, WA, USA) and blinded (AntRenamer, Antoine Potten). HE-stains were evaluated following manual definition of infiltrates. The remainder of stains was evaluated using ImageJ after defining thresholds. The grey matter was excluded from the analysis.

### qPCR Analysis

RNA was isolated from lumbar spinal cord samples with the Qiagen mini Kit according to the manufacturer instructions (Qiagen, Hilden, Germany). Amount and purity of isolated RNA was revealed through nanodrop measurements. Primers were designed using Primer Blast with refseq codes (National Center for Biotechnology Information, Bethesda, USA) for suitable targets, synthesized (microsynth, Balgach, CH) and analyzed for efficiency (**Additional Table 2**). Only primers with an efficiency between 85% and 115% were used. Tata Box protein (Tbp) and Hypoxanthine-guanine-phosphoribosyltransferase 1 (Hprt1) were used as housekeeping genes. Data were generated using a QuantStudio 3 RT-PCR System and analyzed using QuantStudio Design & Analysis Software v1.5.1 (Applied Biosystems, Thermo Fisher Scientific, Waltham, MA, USA).

## Results

### Clozapine Regulates Iron-Mediated Effects of Microglia *In Vitro*

**Figure 1:**
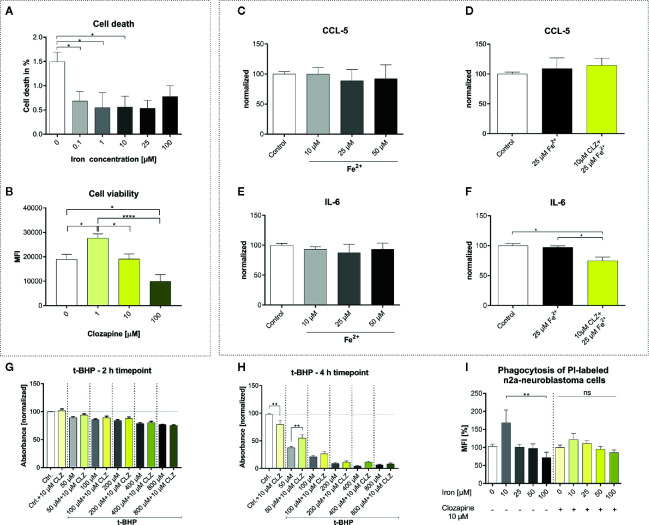
Clozapine enhances microglial viability, reduces the release of IL-6, protects microglia against oxidative stress and normalizes microglial phagocytosis. **(A)** Iron treatment reduced cell death of microglia (cell line HMC3) in low concentrations. **(B)** Viability of microglia was increased upon treatment with clozapine in a concentration of 1 µM (p < 0.05), while concentrations of 100 µM were toxic (p < 0.05). Release of CCL5 **(C)** was not altered after iron treatment (25 µM) **(D)**. Clozapine, however reduced IL-6 release in iron treated microglia **(F)**, while iron itself did not have any effect on IL-6 release **(E)**. **(G)** Oxidative stress, induced by the addition of t-BHP, led to a dose-dependent reduction of cell viability after 2 h; clozapine had no effect after 2 h. **(H)** After 4 h, toxic effects of t-BHP were more pronounced and clozapine was able to attenuate cell death in t-BHP treated cells treated in a dosage of 50 µM (p < 0.05). **(I)** Microglia were treated with iron following addition of dead neurons. Iron in a concentration of 10 µM trended towards enhanced phagocytosis, 100 µM trended towards the opposite. The effects were normalized after clozapine treatment in a dosage of 10 µM. **(A, B)** Data are shown as mean ± SEM of 3 **(A)** and 4 **(B)** independent experiments performed in triplicates **(A)** and quadruplicates **(B)**, **(C–F)** mean ± SEM of 2 independent experiments performed in triplicates, **(G, H)** 3 (2) independent experiments performed in quadruplicates and **(I)** 8 independent experiments for control and 3 independent experiments with clozapine in triplicates and quadruplicates. Data were analyzed using a one-way ANOVA with Tukey’s (A-B, I), non-parametric Kruskal-Wallis with Dunn’s **(C–F)** and Sidak’s **(G–H)** multiple comparison as *post hoc* analysis. **(I)** Outliers were eliminated with ROUT method Q = 1. * = p < 0.05, ** = p < 0.01, **** = p < 0.0001.

To understand the effect of iron release on microglial functions and whether those are altered by clozapine we performed extensive experiments *in vitro*. HMC3 cells were treated with iron in different concentrations and analyzed regarding cell death. Unexpectedly, iron treatment reduced cell death in HMC3 cells in all dosages and did not have a toxic effect up to 100 µM (**Figure 1A**). Clozapine increased microglial viability at a dosage of 1 µM (p <0.05; **Figure 1B**), while concentrations of 100 µM were toxic and reduced microglial viability (p <0.0001; **Figure 1B**). We investigated the release of the chemokine CCL5 and inflammatory cytokine IL-6 to understand effects regarding markers of inflammation. While 25 µM iron supplementation did not alter the cytokine release, pre-treatment with clozapine in a dosage of 10 µM reduced the release of IL-6 following iron treatment by 23% compared to the iron treated control condition (p <0.05; **Figures 1C–F**). Since oxidative stress is a driver of progression, leading to an altered function of both microglia and neurons (4, 14) and since we have shown that clozapine is a potent anti-oxidative compound with a gallic-acid equivalent of 4.6 (p <0.05) (7), we set out to analyze the effect of oxidative stress on microglia and investigated microglial viability upon t-BHP treatment at different time points. Microglial viability was dose-dependently reduced with a reduction of 22% upon treatment with 800 µM after 2 h (**Figure 1G**). Clozapine had no effect after 2 h. Toxic effects of t-BHP treatment were even more pronounced after 4 h (**Figure 1H**). Of note, after 4 h clozapine rescued microglia if treated with 50 µM t-BHP (p <0.01; **Figure 1H**). To analyze whether microglial function is also attenuated we set out to investigate effects on phagocytosis. While iron administration in low concentrations (10 µM) increased phagocytosis, higher concentrations of 100 µM impaired microglial function as indicated by a reduction of phagocytosis of 28% which however lacked significance (**Figure 1I**). Clozapine normalized those effects. Altogether, those data show that clozapine can moderately regulate microglial inflammatory responses and function elicited by iron treatment.

### Clozapine Positively Attenuates Chronic Experimental Autoimmune Encephalomyelitis in a Dose-Dependent Manner

**Figure 2:**
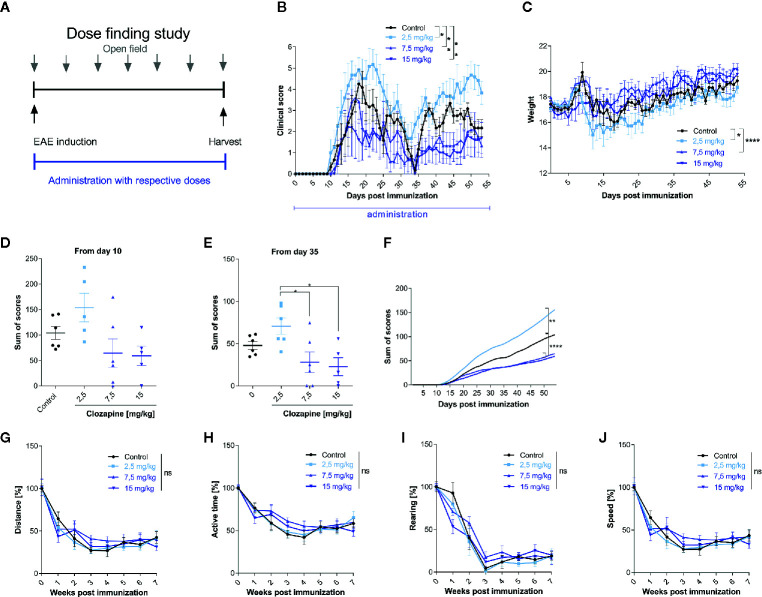
Treatment with clozapine ameliorates chronic EAE dose-dependently in a prophylactic treatment paradigm. **(A)** MOG-immunized C57BL6/J mice (female, 8 weeks old) were treated with different clozapine concentrations (2.5 mg/kg, 7.5 mg/kg, 15 mg/kg) or 5% DMSO/0.0025% acetic acid in saline (control) once a day from day 0 and evaluated weekly using the open field analysis. **(B)** The clinical scores and **(C)** weight of mice treated with 2.5 mg/kg clozapine declined (p = 0.0228 for score, p = 0.0357 for weight) whereas the groups treated with 7.5 mg/kg (p=0.0059 for score, p = < 0.0001 for weight) and 15 mg/kg clozapine (p = 0.0016 for score) improved compared to the control group. **(D)** Differences in sum of scores were not significant regarding the timespan of clinical signs from day 10. **(E)** Sum of scores during the chronic phase of EAE (from day 35) differed significantly for 7.5 mg/kg (p = 0.0222) and 15 mg/kg (p = 0.0136) treated groups compared to the 2.5 mg/kg treated group. **(G–J)** Open field experiments were conducted to measure the sedative effect of clozapine. Groups did not differ regarding distance, active time, rearing and speed. Control group, 2.5 mg/kg group and 7.5 mg/kg group n=6, 15 mg/kg group n=5. Data are shown as mean ± SEM. Non-parametric Kruskal-Wallis test **(B–E, G–J)** and ordinary one-way ANOVA **(F)** with 95% confidence interval. Significances are depicted as ^*^p < 0.05, ^**^p < 0.01, ^****^p < 0.001, ns: not significant (p>0.05).

We then set out to investigate the effect of clozapine in chronic EAE. We investigated different dosages of clozapine applied by oral gavage from the day of MOG-immunization (**Figure 2A**) and treated animals over the whole period of the experiment. To assure that mice received equivalent concentrations of clozapine, animals were treated by oral gavage. Pilot experiments showed that the administration of 30 mg/kg clozapine led to exuberant sedation resulting even in death of some animals (not shown). We therefore performed a dose-finding study to establish the effective and safe dose of 15 mg/kg. While control animals treated with vehicle showed marked signs of EAE with a mean score of 4.3 ± 0.7 at the peak of EAE and chronification over more than 50 d, clozapine led to a dose-dependent reduction of clinical signs (**Figure 2B**). Disease incidence declined dose-dependently following clozapine administration (**Additional Table 3**). Moreover, the onset of clinical signs was delayed by 3 d in 15 mg/kg treated mice. While the clinical scores of mice treated with 2.5 mg/kg clozapine increased (p = 0.0228) we observed an improvement in mice treated with 7.5 mg/kg (p = 0.0059) and 15 mg/kg clozapine (p=0.0016) compared to the control condition. This was reflected in a higher body weight as marker of general health with the exception that we could not observe a significant weight change in 15 mg/kg clozapine treated animals (7.5 mg/kg vs. control p <0.0001, 2.5 mg/kg vs. control p = 0.0357; **Figure 2C**). Positive effects of clozapine on the clinical course was reflected in sum-of-scores. While sum-of-scores from day 10 to the end of the experiments only trended towards positive effects of higher clozapine concentrations (**Figure 2D**), analysis of sum-of-scores during the chronic phase from day 35 showed a significant effect of 7.5 mg/kg (28.1 ± 12.1; p <0.05) and 15 mg/kg (22.8 ± 10.6; p<0.05) compared to 2.5 mg/kg (**Figures 2E, F**).

Since clozapine has sedative effects, also documented in our pilot experiments, we wanted to rule out that those might influence the neurological phenotype. We therefore performed an open-field analysis and investigated the overall distance, the active time, rearing and the speed. While we could document a reduction of all aforementioned tests, presumably due to habituation, clozapine treated groups and the control group did not differ (**Figures 2G–J**).

### Therapeutic Administration of Clozapine Also Ameliorates Chronic EAE

**Figure 3:**
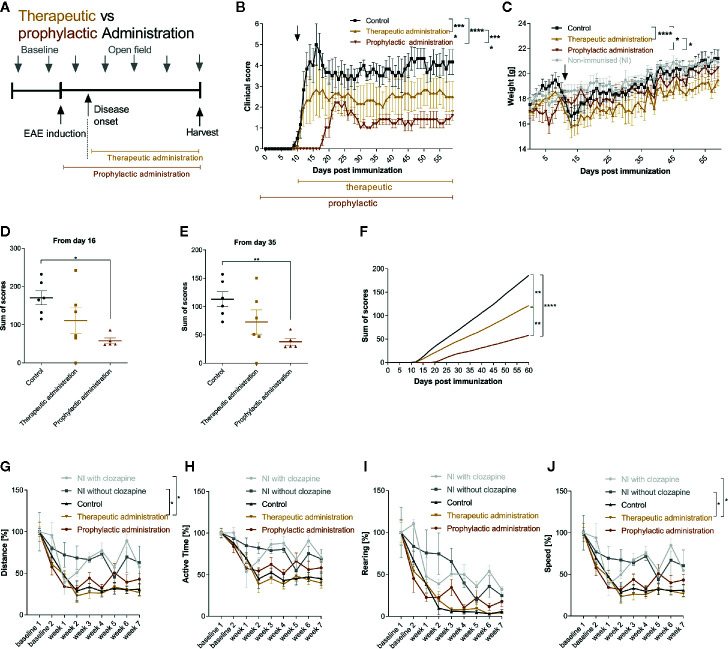
Therapeutic and prophylactic administration ameliorate chronic EAE. **(A)** MOG-immunized C57BL6/J mice (female, 8 weeks old) were treated with 15 mg/kg from day 0 (prophylactic administration) or from day 11 (therapeutic administration, 50% of mice showed clinical signs at this timepoint) once a day. The control group was treated with 5% DMSO/0.0025% acetic acid in saline (vehicle) from day 0. **(B)** The clinical condition and **(C)** weight of the control mice declined compared to the prophylactically (p < 0.0001 for score, p = 0.0202 for weight) and therapeutically treated (p < 0.0001 for score, p < 0.0001 for weight) mice. **(D)** The sums of scores for the timespan of clinical symptoms after peak disease (from day 16) were higher in the control group compared to the prophylactically treated group (p=0.0116). **(E)** This was mirrored during the chronic phase of EAE (p=0.0098) and **(F)** regarding the analysis of the overall sum of scores. **(G–J)** Open field experiments were conducted to measure the sedative effect of clozapine. Speed and distance were significantly higher in non-immunized groups compared to therapeutic treatment. The remainder did not differ regarding distance, active time, rearing and speed. Control group n= 6, therapeutic treatment group n = 6 and prophylactic treatment group n=5. Data are shown as mean ± SEM. Non-parametric Kruskal-Wallis test **(B–E, G–J)** and ordinary one-way ANOVA **(F)** with 95% confidence interval. Significances are depicted as ^*^p < 0.05, ^**^p < 0.01, ^****^p < 0.001.

Having identified the effective dose (15 mg/kg body weight), we set out to investigate whether clozapine also attenuates MOG-EAE if applied in a therapeutic treatment paradigm and compared this setting to a prophylactic paradigm with treatment initiation from the day of immunization. We chose a time-point when about 50% of animals had developed clinical signs of EAE after 11 d (**Figure 3A**) and used the effective dosage of 15 mg/kg clozapine once daily, identified in the dose-finding study. In this experiment, the onset of clinical signs in the prophylactic group was even later compared to the dose-finding study (8 d delay, p <0.0001; **Additional Table 4**). While the control group displayed robust chronification after the initial relapse, both treated groups had reduced signs of disease (prophylactic administration 1.6 ± 0.5 (mean ± SEM); therapeutic administration 2.5 ± 1.8) in contrast to the control group at the end of the experiment (4.2 ± 1.5; p <0.0001 vs. therapeutic administration; p <0.0001 vs. prophylactic treatment) (**Figure 3B**). As expected, the prophylactic treatment paradigm had stronger positive effects and delayed the onset of disease by 8 d compared to the control group. Of note, the weight increase was notably lower in treated mice at the end of the experiment (prophylactic treatment 20.5 ± 1.5 g; therapeutic treatment 19.5 ± 1.2 g) in contrast to the control group (21.2 ± 1.7 g; p=0.02 vs. prophylactic treatment; p <0.0001 vs. therapeutic treatment; **Figure 3C**). Positive treatment effects were again reflected in total sums of scores (day 16: control group 170.7 ± 18.2 vs. prophylactic treatment 58.1 ± 7.2, p = 0.0116; day 35: control group 113.0 ± 13.3 vs. prophylactic treatment 38.0 ± 6.0 p = 0.0098; **Figures 3D–F**). Open field experiments did not show differences between the control group and the two therapeutic regimen. To rule out sedative effects of clozapine, we also investigated non-immunized mice with or without clozapine. While clozapine treated mice were less active in the first measurements, the effect was gone at week 3, arguing for habituation (**Figures 3G–J**).

### Treatment During Late Chronic EAE

**Figure 4:**
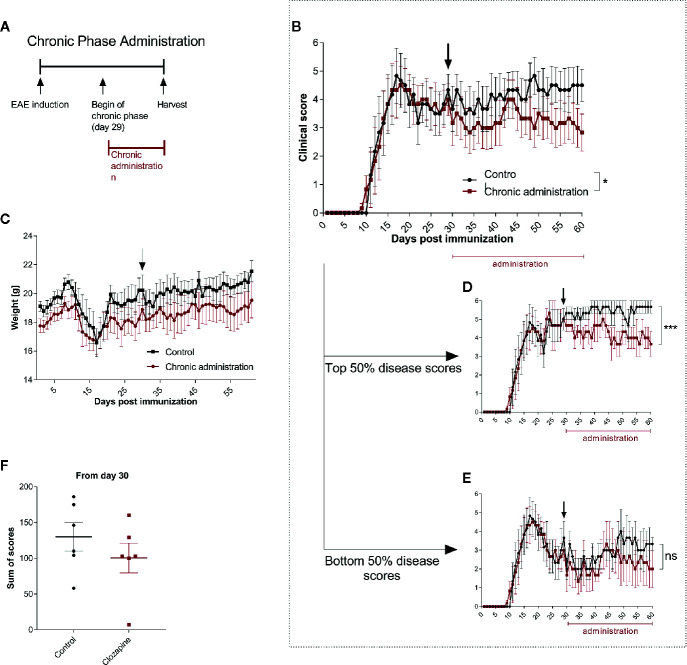
Treatment with clozapine during chronic EAE ameliorates clinical signs with higher benefit in animals with higher disability. **(A)** MOG-immunized C57BL6/J mice (female, 8 weeks old) were treated with 5% DMSO/0.0025% acetic acid in saline (vehicle) from day 0. Animals were randomized from the chronic phase (day 29) and one group was treated with 15 mg/kg clozapine once a day. **(B)** The clinical condition of treated mice improved in the chronic phase compared to the control group (p = 0.0388) while **(C)** weight did not differ. **(D)** This effect was mediated by mice with higher disability (top median) which showed a decline of signs (p = 0.0005) whereas **(E)** the lower median did not profit during the chronic phase (p = 0.1204). **(F)** Sums of scores in the chronic phase did not differ. Control group n = 6, chronic phase treatment group n = 6. Data are shown as mean ± SEM. Area under the curve (AUC) with unpaired t-test **(B–E)** and Mann-Whitney test **(F)** with 95% confidence interval. Significances are depicted as ^*^p < 0.05, ^***^p < 0.001, ns: not significant (p>0.05).

Having established that clozapine is effective also in a therapeutic treatment paradigm we asked whether late treatment might still be effective and therefore performed analyses of clozapine treatment in late chronic-EAE. To investigate this phase, we performed an experiment with therapy induction from day 29 (**Figure 4A** and **Additional Table 5**). Here, we could document mild beneficial effects (mean score at day 60: control group 4.3 ± 0.7, clozapine group 2.8 ± 0.7; p <0.05, **Figure 4B**). Of note, especially mice with a higher disease activity (top 50% of the scores) profited from the medication (**Figure 4D**, p <0.001), whereas mildly impaired animals did not show a response (**Figure 4E**). Clozapine had no effect on weight. Sum of scores during the chronic phase therefore did not differ (control 129.8 ± 19.7; clozapine treatment 100.3 ± 20.9; p = 0.2229).

### Histological Analysis Shows Reduced Infiltration and Demyelination

**Figure 5:**
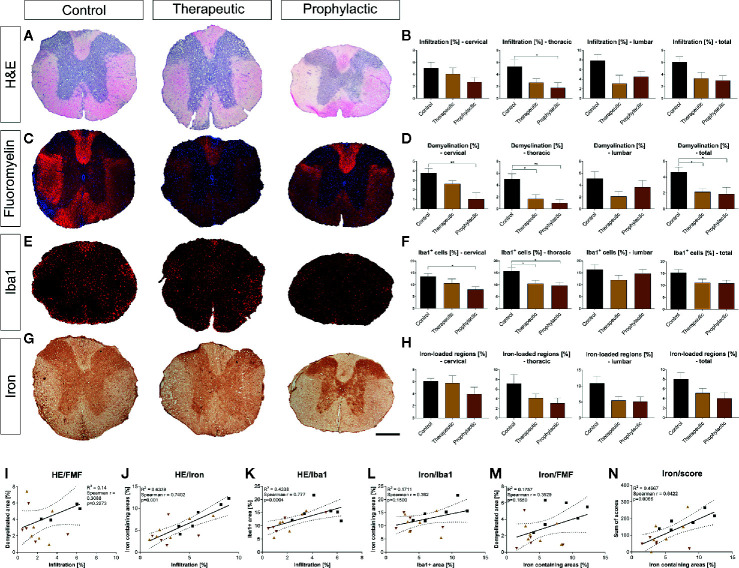
Prophylactic and therapeutic clozapine administration reduce infiltration of inflammatory cells, iron loaded regions, demyelination and microglia activation in spinal cord sections. **(A)** Representative images of H&E stained spinal cord sections. **(B)** Clozapine administration led to a trend towards decreased infiltration in all spinal cord segments, reaching significance in thoracic segments of the spinal cord in prophylactically treated mice (p=0.0427). **(C)** Representative images of fluoromyelin stained spinal cord sections. **(D)** Demyelination of total spinal cord sections was significantly reduced after both symptomatic (p < 0.05) and therapeutic administration (p < 0.05). **(E)** Infiltration of macrophages/microglia, as assessed using Iba1 staining. **(F)** Less infiltration in cervical and thoracic cord in prophylactic treated mice (p < 0.05), lacking significance upon analysis of the whole spinal cord. **(G)** Representative images of iron stained sections. **(H)** Trend towards less iron deposition in all sections, lacking significance. **(I)** While infiltration and demyelination as well as **(L)** iron deposition and macrophage/microglial infiltration and **(M)** iron deposition and demyelination did not correlate, **(J)** there was a strong correlation of infiltration and iron deposition (Spearman r = 0.74; p = 0.001), **(K)** general infiltration and microglial/macrophage infiltration (Spearman r = 0.77; p = 0.0004) as well as (N) iron deposition and individual animal score (Spearman r = 0.64; p = 0.0066). **(B, D, F, H)** Ordinary one-way ANOVA and Dunnett’s multiple comparison with a single pooled variance and 95% confidence interval. **(I-N)** Correlation using Spearman r and R^2^. Data are shown as mean ± SEM. Significance is shown as *p < 0.05, **p < 0.01. Scale bar for a, c, e and g is 400 µm.

Histological analysis of the spinal cord revealed that general infiltration in all parts of the spinal cord trended towards a reduction in the prophylactic group compared to the control condition (thoracic cord p <0.05; **Figures 5A, B**). Demyelination was significantly reduced both in the symptomatic and prophylactic treatment group compared to the control (p <0.05, **Figures 5C, D**). Microglia were also reduced in the cervical and thoracic cord (p <0.05), which, however, lacked significance upon analysis of the whole spinal cord (**Figures 5E, F**). We then evaluated the effects of clozapine on iron deposition *in vivo*. Again, we saw a reduction, mostly in the prophylactic group, which however lacked significance (**Figures 5G, H**). Correlations of histological data showed that general infiltration and demyelination, iron deposition and infiltration of Iba1^+^ cells as well as iron deposition and demyelination did not correlate (**Figures 5I, L, M**), while general infiltration and iron deposition (r = 0.74, p = 0.001) as well as general infiltration and infiltration of Iba^+^ cells (r = 0.78, p = 0.0004) strongly correlated (**Figures 5J, K**). Moreover, iron deposition and sum of scores strongly correlated (r = 0.64, p = 0.0066) (**Figure 5N**).

### Transcription of Iron Metabolism Proteins and Markers of Inflammation Are Regulated by Clozapine

**Figure 6:**
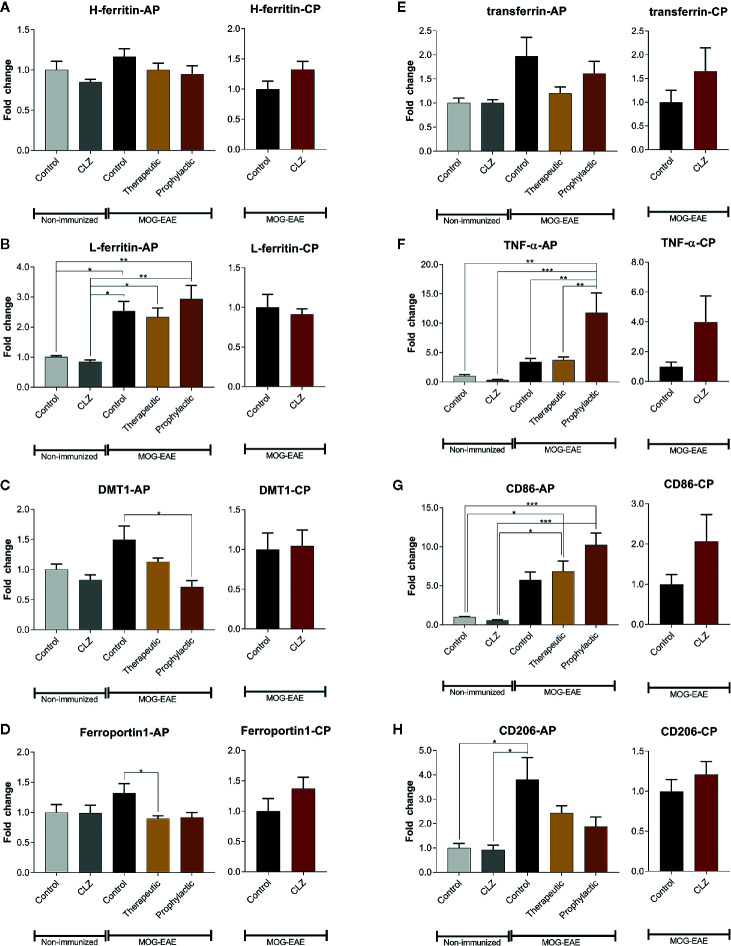
Clozapine administration alters iron metabolism of the spinal cord. **(A)** While H-ferritin was not affected, **(B)** L-ferritin was upregulated in EAE mice (p < 0.05) but not altered following clozapine administration. **(C)** DMT-1 was downregulated following prophylactic clozapine administration compared to untreated EAE mice (p < 0.05). **(D)** Ferroportin-1 was downregulated following therapeutic treatment with clozapine (p < 0.05). **(E)** Transferrin was not affected. **(F)** Prophylactic clozapine administration led to significant upregulation of TNF-α compared to non-immunized mice or therapeutic treatment (p < 0.01). **(G)** CD86 was significantly upregulated compared to non-immunized mice (p < 0.05), but lacked significance compared to EAE mice. **(H)** CD206 was upregulated in vehicle EAE mice, clozapine had no effect. Non-immunized groups n = 3 each, acute phase treatment groups n = 6 each (prophylactic administration n = 5) and chronic phase treatment groups n = 6 each. Data were normalized to non-immunized and untreated mice for acute phase treatment and to vehicle group for chronic phase treatment. Tbp and Hprt1 were used as housekeeping genes. Data are shown as mean ± SEM. One-way ANOVA with Tukey’s multiple comparison as *post hoc* analysis (AP) and two tailed unpaired t-test (CP) were used for analysis. Significances are depicted as ^*^p < 0.05, ^**^p < 0.01 ^***^p < 0.001.

Since clozapine reduces iron load upon clozapine treatment as evidenced using histological analyses, we further elucidated regulation of proteins involved in iron metabolism (**Figure 6**). H-ferritin was unaffected in EAE mice and upon clozapine treatment. L-ferritin was significantly upregulated in EAE mice (p <0.05), but not affected by clozapine treatment. Treatment with clozapine during the acute phase led to decreased DMT-1 transcription (vehicle vs. prophylactic p=0.0031). Ferroportin 1 was downregulated upon therapeutic administration (p <0.05). Treatment with clozapine during the chronic phase did not affect transcription of aforementioned proteins. TNF-α as inflammatory marker was significantly upregulated upon prophylactic therapy (p <0.01) compared to both the vehicle group and therapeutic therapy. CD86 trended towards an increase following clozapine therapy compared to vehicle treated EAE mice. CD206 was significantly upregulated in vehicle EAE mice compared to non-EAE mice and trended towards a downregulation following clozapine therapy which lacked significance.

### Clozapine Induced Modest Effects on Peripheral Immune Cells

To understand effects of clozapine on peripheral immune cell subsets we investigated immune cells changes in blood, spleen and lymph node cells. Clozapine significantly reduced the frequency of CD4^+^ T cells in all compartments with strongest and dose-dependent effects in lymph nodes (15 mg/kg 29% reduction, p <0.05; Figure e1). Th17 (CD4^+^IL17^+^) cells were reduced in the spleen (p<0.05). Th1 (CD4^+^IFNγ^+^) cells were not considerably affected, in lymph nodes a slight increase was seen in 15 mg/kg clozapine treated mice which lacked significance. Clozapine interestingly induced a profound reduction of regulatory (CD4^+^CD25^+^FoxP3^+^) T cells in the blood in 2.5 mg/kg clozapine treated mice (p <0.01) as well as dose-dependently in lymph nodes (p <0.05). CD86^+^ antigen-presenting cells were reduced in the spleen (p <0.05), while activated CD86^+^F4/80^+^ cells did not differ. Clozapine had no effects on CD8^+^ cytotoxic T cells or CD45R^+^ B cells.

## Discussion

Therapeutic approaches to target aspects of progressive MS are still not effective enough to halt disease progression in most patients. This can in part be explained by the plethora of mechanisms being involved in progression, amplifying themselves *vice versa* (4). By employing biochemical assays, we identified a group of neuroprotective generic medications with a well-known safety profile and potential for therapeutic development in progressive MS. We now investigated one of the medications identified, the antipsychotic clozapine, using *in vitro* experiments regarding its ability to modify microglial activation and foster neuroprotection in an animal model of progressive disease. In culture, clozapine moderately reduced the release of IL-6 of iron treated microglia and increased microglial viability in low concentrations. Moreover, iron impaired microglial phagocytosis was regulated using clozapine. *In vivo*, clozapine reduced disability progression in chronic EAE dose-dependently both in a prophylactic and therapeutic scenario.

Until now, the effectiveness of therapeutics for progressive MS is not overwhelming. A different approach than developing and designing new therapeutics against progression is tackling disease pathomechanisms with already approved generic medications. An advantage of this avenue is that medications are authorized for another indication, they have a well-known safety profile due to years of clinical practice and therefore a fast translation into clinical trials is potentially feasible. Moreover, those medications are also affordable for countries with poor healthcare systems. Clozapine is a low molecular weight atypical antipsychotic and follows Lipinski’s rule, providing its exceptional ability to penetrate the CNS (15). It binds to different receptors such as dopamine, serotonin and alpha- and muscarinic acetylcholine receptors (15).

Since clozapine elicits sedative effects, we aimed to rule out that those might interfere with general health in treated mice. We therefore performed extensive open field analyses, which showed that initially observed sedative effects vanish within two weeks of treatment. For translation into human it is essential that dosages applied in mice can also realistically be achieved in human without inducing side effects. The concentrations of 2.5 – 15 mg/kg in mice used in our experiment are equivalent to human dosages between 0.2 and 1.2 mg/kg clozapine per day (16), equivalent to 14–84 mg for a 70-kg individual. Most patients treated for schizophrenia receive dosages ranging from 200-450 mg/day with a maximum of up to 900 mg/kg. Adverse effects occur especially at dosages above 450 mg/kg (15). Since we already observed positive effects in equivalent dosages of 84 mg, reaching a fraction of concentrations usually used for schizophrenia, we assume that the concentrations used in our experiments would be both clinically feasible and effective in patients with progressive MS; even if it might be suggested that side effects already occur in patients with progressive MS using lower dosages.

We provided evidence that clozapine is neuroprotective against iron mediated neurotoxicity, leading to the preservation of about 100% of neurons after a 24 h treatment period with iron in culture (7). Clozapine moreover is mitochondrioprotective (p <0.0001) and has antioxidative effects with an gallic acid equivalent of 4.6 (p <0.05), a potent anti-oxidative compound (7). Of note, we did not observe effects on T lymphocyte proliferative activity. Clozapine has been investigated in EAE previously (17, 18). Green et al. showed that clozapine has greater efficacy in halting EAE than risperidone, quetiapine or olanzapine (18). The administration of clozapine was, however, achieved by addition to the chow. The strong initial sedative effects of clozapine, documented in our experiments presented here, suggest, that the way of application might have led to incongruency due to reduced uptake. Clozapine does not reduce demyelination in the toxic demyelination cuprizone model but enhances the rate of functional recovery therein, associated with reduced astrocytic and microglial activation (19). Microglial activation is a key contributor to chronic inflammation in progressive MS and is therefore target for therapeutic development. Since microglia elicit not only negative but also protective effects, microglial function should be altered and not arrested. Our findings indicate that clozapine modulates microglial activity by regulating the inflammatory effect of free iron regarding phagocytosis of dead neurons, release of the inflammatory cytokine IL-6 and viability after oxidative stress. HMC3-microglia express NOX4 which leads to a constitutive generation of ROS, inducing an expression of IL-6 mRNA (20). While we could not observe an increase of IL-6 with ferrous iron, the decrease of IL-6 release following clozapine treatment might be due to a downregulation of the NOX4 system with reduced ROS-decrease. Those data are in line with reports showing that clozapine reduces the release of NO in LPS-treated microglia (21). Effects of clozapine on microglia might in part be mediated by calcium/calmodulin dependent Akt activation (22). It cannot be ruled out that the strong effects in EAE might also in part be mediated by immunomodulatory effects of clozapine. While we did not observe effects on the proliferation of T cells in our systematic screening (7), it is known that clozapine has strong effects on immune cells with reduction in class-switched memory B cells and secondary antibody deficiency (23), which might have been a contributing factor in our experiments.

Iron overload is a hallmark of the ageing CNS and is associated with several neurodegenerative disorders (24). In MS, iron has both beneficial and detrimental effects (8). Iron deposition in the basal ganglia correlates with progression and excess iron is toxic since it drives oxidative stress *via* the Fenton reaction (8). On the other hand, iron is important for the viability of oligodendrocytes and those receive their trophic support of iron in the form of H-ferritin through microglia (25). Iron metabolism is tightly regulated through a number of mechanisms and proteins. The upregulation of iron importer DMT-1 and downregulation of iron exporter ferroportin1 is a consequence of inflammatory stimuli and *vice versa* (26, 27). Transferrin is able to buffer iron (28) but can also be rapidly effluxed from the brain to the blood (29). H-ferritin has a ferroxidase activity and can catalyze the oxidation of ferrous iron to ferric iron by consuming the substrates for the Fenton’s reaction; L-ferritin mediates and acclererates its storage (28, 30). Our data suggest that clozapine attenuates the uptake of iron by downregulation of DMT-1, leading to reduced iron in the spinal cord, evidenced by the histological analyses. Downregulation of ferroportin1 suggests a compensatory mechanism to prevent further iron loss. Late treatment during the chronic phase did not have an effect on DMT-1. Since we could also document reduced demyelination in early clozapine treated EAE mice we assume that the dosage used in our experiments did not elicit deleterious effects on oligodendrocytes. Of note, we could document an upregulation of TNF-α, an unexpected finding in light of the strong anti-inflammatory properties of clozapine. TNF exists as transmembrane form with signaling through TNFR2 and TNFR1 and a soluble form which acts *via* TNFR1 (31). Oligodendroglial TNFR2 is a key mediator of transmembrane TNF dependent protection in EAE, crucial for oligodendrocyte differentiation (31). TNF-α also exhibits anti-inflammatory effects on TGF-β treated APCs, mediated by the TNF-R2 and thereby regulating immune responses (32). Those data altogether suggest, that the upregulation of TNF-α by clozapine mirrors the anti-inflammatory effects of the medication. To understand effects on innate immune cells we also investigated transcriptional changes of CD206 and CD86. CD206 peaks in late active and inactive MS lesions (33); the downregulation mediated by clozapine could thus in part be due to reduced lesion load following therapy. This is supported by data from the chronic experiment showing slight (but not significant) upregulation of CD206 following clozapine treatment, indicating enhanced regeneration.

There are some limitations of the data presented here. First, there is not an optimal model mimicking all aspects of progressive MS (34) including chronic EAE in C57Bl6 mice used here. Other models, previously used by us and others include the Biozzi Abh mouse model (35) which suffers from inconsistent EAE induction (36), the NOD model which is difficult to induce in our hand, or models using the Theiler murine virus. To address this question we performed extensive initial cell culture screening and addressed specific quesions using cell culture models (7). The initial screening also has limitations such as the usage of a circumscribed number of generic medications, used in a single screening concentration of 10 µM (7). Another drawback is the use of cell lines, which, however, enables performing complex experiments with several conditions, as done by us. Moreover we examined transcription changes of the whole spinal chord and did not perform single-cell RNA sequencing, which would have helped to better evaluate alterations induced by clozapine on different cell types, a question worthwhile to adress in future experiments. While the effect of the prophylactic and therapeutic treatment paradigm was strong, the effect of a treatment during the chronic phase was, although significant, less robust, and driven by highly impaired mice as identified following a post-hoc analysis. Moreoever, clozapine as substance has drawbacks such as (initial) sedative effects and agranulocytosis; hence, patients would have to be monitored closely to minimize the risks of the medication.

## Conclusion

In summary, the work presented here shows that clozapine regulates microglial function upon iron stimulation, reflected in reduced release of inflammatory cytokines and normalization of neuronal phagocytosis, a scenario relevant in patients with progressive MS. Clozapine moreover dose-dependently attenuates clinical signs in chronic EAE, even if applied late during the chronic stage of the disease, with positive effects on histological markers such as demyelination. Dosages applied *in vivo* reflect low dosages readily achievable in human. We therefore consider clozapine as interesting target molecule for further development as add-on therapy in progressive MS.

## Glossary

EAE: Experimental autoimmune encephalomyelitis; DMT-1 : Divalent metal transporter 1; MS: Multiple sclerosis; RRMS: Relapsing-remitting multiple sclerosis; SPMS: Secondary progressive multiple sclerosis; PPMS: Primary progressive multiple sclerosis; BBB: Blood-Brain-Barrier; CNS: Central nervous system; HMC3: human microglial clone 3 cell line; PI: Propidium iodide; t-BHP: *tert*-Butyl hydroperoxide; ns: not significant; MTT: 3-(4,5-dimethylthiazol-2-yl)-2,5-diphenyltetrazolium bromide; MOG: Myelin oligodendrocyte glycoprotein; TNF-R1/R2: Tumor necrosis factor receptor 1/2.

## Data Availability Statement

The original contributions presented in the study are included in the article/**Supplementary Material**. Further inquiries can be directed to the corresponding author.

## Ethics Statement

The animal study was reviewed and approved by animal care committee of North Rhine-Westphalia, Germany (LANUV, no. 84-02.04.2017.A132).

## Author Contributions

UC: Investigation and acquisition of data, analysis and interpretation of data, visualization, study concept or design, and original draft of the manuscript. SH: Investigation and acquisition of data, analysis and interpretation of data, and revising the manuscript. LK: Investigation and acquisition of data, analysis and interpretation of data, and visualization. JD: Investigation and acquisition of data, analysis and interpretation of data, and revising the manuscript. BA: Investigation and acquisition of data, analysis and interpretation of data, and revising the manuscript. RG: Analysis and interpretation of data, revising the manuscript, and funding acquisition. SF: Analysis and interpretation of data, visualization, original draft and reviewing of the manuscript, funding acquisition, and study supervision. All authors contributed to the article and approved the submitted version.

## Funding

This study was supported by the Medical Faculty of Ruhr-University Bochum (FoRUM program F898N2-2017) to SF.

## Conflict of Interest

SF filed a United States Patent Application relating and entitled to “Treatment for Progressive Multiple Sclerosis”, No. 62/412,534, filed October 25, 2016.

The remaining authors declare that the research was conducted in the absence of any commercial or financial relationships that could be construed as a potential conflict of interest.
