# Supplementary material for: Clozapine Regulates Microglia and Is Effective in Chronic Experimental Autoimmune Encephalomyelitis
Source: Front Immunol. 2021 May 3;12:656941. doi: 10.3389/fimmu.2021.656941 (PMC8126707; doi:10.3389/fimmu.2021.656941)
Supplement: Supplementary file 1 [file DataSheet_1.docx]

**ADDITIONAL MATERIAL**

**Clozapine regulates microglia and is effective in a model of progressive multiple sclerosis**

Ulaş Ceylan^1^, Steffen Haupeltshofer^1^, Laura Kämper^1^, Justus Dann^1^, Björn Ambrosius^1^, Ralf Gold^1^ and Simon Faissner^1^

Department of Neurology, Ruhr-University Bochum, St. Josef-Hospital, Germany

Corresponding author

Ass. Prof. Dr. Simon Faissner, MD

Department of Neurology

Ruhr-University Bochum, St. Josef-Hospital

Gudrunstr. 56, 44791 Bochum, Germany

Tel: +49-234-5092411; Fax: +49-234-5092740

Email: [simon.faissner@rub.de](mailto:simon.faissner@rub.de)

**Additional figure**

**Additional figure 1: Effects on peripheral immune cell subsets.** Clozapine significantly reduced CD4 positive cells in blood (A), spleen (B) and lymph nodes (C) with strongest effects in lymph nodes. In spleen, Th17 cells were reduced after 15 mg/kg clozapine treatment. Th1 cells were not affected. (A) Regulatory T cells were strongly reduced in blood in 2.5 mg/kg clozapine treated mice as well as (C) dose-dependently in lymph nodes. CD86^+^ antigen-presenting cells were reduced in the spleen following treatment with 15 mg/kg clozapine without effect on activated cells. Cytotoxic T cells and B cells were not affected. Data were analyzed using a one-way ANOVA with Dunnett`s multiple comparison as post hoc analysis: *=p<0.05, **=p<0.01.

**Additional tables**

**Additional table 1**

(A) Antibodies used for flow cytometry

| **Antibody** | **Vendor** | **Catalogue number** |
| --- | --- | --- |
| Anti-Mouse CD45R PE-Cy7 | BD Pharmingen | 552772 |
| Anti-Mouse CD8a PE | BD Pharmingen | 561095 |
| Anti-Mouse CD86 FITC | BD Pharmingen | 553691 |
| Anti-Mouse F4/80 APC | Invitrogen | 17-4801-82 |
| Anti-Mouse CD4 FITC | Invitrogen | 11-0042-82 |
| Anti-Mouse CD25 APC | Invitrogen | 17-0251-82 |
| Anti-Mouse FoxP3 PE | Invitrogen | 12-5773-82 |
| Anti-Mouse CD4 APC | Invitrogen | 17-0042-82 |
| Anti-Mouse IFN gamma FITC | Invitrogen | 11-7311-82 |
| Anti-Mouse IL-17 PE | Invitrogen | 12-7177-81 |

(B) Antibodies used for histology

| **Antibody** | **Dilution** | **Species** | **Vendor** | **Catalogue number** |
| --- | --- | --- | --- | --- |
| anti-Iba-1 | 1:500 | Rat | FUJIFILM Wako Pure Chemical Corporation, Osaka, Japan | 019-19741 |
| goat-anti-rabbit-Alexa Fluor 568 | 1:1000 |  | abcam, Cambridge, UK | ab175471 |
| FluoroMyelin | 1:300 |  | Invitrogen | F34652 |

**Additional table 2**

Primer sequences

| **Primer name** | **Sequence** |
| --- | --- |
| ferritin light polypeptide 1 (Ftl1) | F: 5´- CGG AGG GTC AAC ATG CTA TAA -3´  R: 5´- GGA AGC GAG TAC AGT GGG AA -3´ |
|  | F: 5´- CAT CTC CTC GCT GCC TTC AG -3´  R: 5´- AGA AGC CCA GAG AGA GGT AGG -3´ |
| mannose receptor, C type 1 (Mrc1) | F: 5´- AAC CAG TTC CTT GAG CTC GG -3´  R: 5´- CTG ATT AGG GCA GCC GGT AG -3´ |
|  | F: 5´- GTC AGA ACA GAC TGC GTG GA -3´  R: 5´- AGG GAT CGC CTG TTT TCC AG -3´ |
| CD86 antigen (Cd86) | F: 5´- CTT ACG GAA GCA CCC ACG AT -3´  R: 5´- TGT AAA TGG GCA CGG CAG AT -3´ |
| ferritin heavy polypeptide 1 (Fth1) | F: 5´- CAG AAC TAC CAC CAG GAC GC -3´  R: 5´- TCA GAG CCA CAT CAT CTC GG -3´ |
| tumor necrosis factor (Tnf) | F: 5´- ATG GCC TCC CTC TCA TCA GT -3´  R: 5´- TGG TTT GCT ACG ACG TGG G -3´ |
| solute carrier family 11  (DMT-1) | F: 5´- AAA GAT GCC AGA CGA TGG CG -3´  R: 5´- ATC CGT GGG ACC TTG GGA TA -3´ |
| transferrin (Trf) | F: 5´- AGC CGA TGC TAT GAC CTT GG -3´  R: 5´- ACT GCC CGA GAA GAA ACT GG -3´ |
| solute carrier family 40 (ferroportin1) | F: 5´- GGC ACT TTG CAG TGT CTG TG -3´  R: 5´- GTG ACG TCT GGG CCA CTT TA -3´ |
| TATA box binding protein (Tbp) | F: 5´- AGC TCT GGA ATT GTA CCG CA -3´  R: 5´- TGA CTG CAG CAA ATC GCT TG -3´ |
| hypoxanthine guanine phosphoribosyl transferase (Hprt) | F: 5´- ACA GGC CAG ACT TTG TTG GA -3´  R: 5´- ACT TGC GCT CAT CTT AGG CT -3´ |

**Additional table 3: Clinical data related to the dose-finding EAE (Figure 2).**

|  |  |  | Clozapine doses | | |
| --- | --- | --- | --- | --- | --- |
|  |  | Control | 2,5 mg/kg | 7,5 mg/kg | 15 mg/kg |
| Disease incidence (n) | | 100% (6) | 100% (6) | 83% (5) | 67% (4) |
| Death related to EAE (n) | | 0% (0) | 0% (0) | 0% (0) | 0% (0) |
| Peak clinical score  (mean ± SEM) | | 4.3 (0.7) | 5.2 (0.7) | 3.6 (1.1) | 2.4 (1) |
| Onset of signs [day]  (mean ± SEM) | | 13.3 (1.2) | 12.3 (0.9) | 13.4 (0.8) | 16.5 (1.8) |
| Sum of scores from day 10 (mean ± SEM) | | 104 (12.9) | 155.7 (22.9) | 64.5 (27.7) | 59.2 (18.9) |
| Sum of scores from day 35 (mean ± SEM) | | 47.9 (4.7) | 70.7 (9.6) | 28.1 (12.1) | 22.8 (10.6) |
| Death related to other reason apart from EAE (n) | | 0% (0) | 0% (0) | 0% (0) | 17% (1) |

**Additional table 4: Clinical data related to the therapeutic EAE (Figure 3).**

|  |  |  | 15mg/kg Clozapine | |
| --- | --- | --- | --- | --- |
|  |  | Control | Therapeutic | Prophylactic |
| Disease incidence (n) | | 100% (6) | 83% (5) | 100% (6) |
| Death related to EAE (n) | | 0% (0) | 0% (0) | 0% (0) |
| Peak clinical score  (mean ± SEM) | | 5 (1) | 2.8 (0.7) | 2.2 (0.2) *P=0.0206 (*)* |
| Onset of signs [day]  (mean ± SEM) | | 11.2 (0.6) | 13.2 (1.6) | 19.4 (0.7) *P<0.0001 (****)* |
| Sum of scores from day 16  (mean ± SEM) | | 170.7 (18.2) | 110.7 (34.3) | 58.1 (7.2) *P=0.0002 (***)* |
| Sum of scores from day 31  (mean ± SEM) | | 113 (13.3) | 72.8 (21.4) | 37.9 (5.7) *P=0.0004 (***)* |
| Death related to other reason apart from EAE (n) | | 0% (0) | 0% (0) | 17% (1) |

**Additional table 5: Clinical data related to EAE with treatment during the chronic phase (Figure 4).**

|  | Control | Chronic phase treatment  15 mg/kg clozapine |
| --- | --- | --- |
| Disease incidence (n) | 100% (6) | 100% (6) |
| Death related to EAE (n) | 0% (0) | 0% (0) |
| Peak clinical score  (mean ± SEM) | 4.8 (0.7) | 4.5 (0.7) |
| Onset of signs [day]  (mean ± SEM) | 12.8 (1.5) | 11.3 (1.4) |
| Sum of scores from day 0 to 29 (mean ± SEM) | 72.8 (15.5) | 72.8 (14.4) |
| Sum of scores from day 30 (mean ± SEM) | 129.8 (19.7) | 100.3 (20.9) |
| Death related to other reason apart from EAE (n) | 0% (0) | 0% (0) |
